# Polymorphisms of *IL23R* and Fuchs’ syndrome in a Chinese Han population

**Published:** 2010-12-05

**Authors:** Hongyan Zhou, Zhengxuan Jiang, Peizeng Yang, Shengping Hou, Fuzhen Li, Qinmeng Shu, Yuanyuan Chen, Feilan Chen

**Affiliations:** 1 Zhongshan Ophthalmic Center of Sun Yat-sen University, Guangzhou, P.R. China; 2 The First Affiliated Hospital of Chongqing Medical University, Chongqing Key Laboratory of Ophthalmology, and Chongqing Eye Institute, Chongqing, P.R. China

## Abstract

**Purpose:**

The aim of the study was to investigate the association of polymorphisms of the interleukin-23 receptor (*IL23R*) gene with Fuchs’ syndrome in a Chinese Han population.

**Methods:**

Three single-nucleotide polymorphisms (SNPs), rs7517847, rs11209032 and rs17375018 of *IL23R* were genotyped in 138 Chinese Han patients with Fuchs’ syndrome and 407 healthy controls by using the polymerase chain reaction-restriction fragment length polymorphism (PCR-RFLP) method. Data were analyzed by χ^2^ analysis.

**Results:**

All genotype and allele distributions in patients with Fuchs’ syndrome and healthy controls were in Hardy–Weinberg equilibrium. The frequency of the rs11209032 AA genotype was significantly increased in patients with Fuchs’ syndrome as compared to controls (corrected p [pc]=0.036, OR 1.86, 95%CI 1.21 to 2.86). There were no statistically significant differences between patients and healthy controls concerning the other two tested SNPs (rs17375018 and rs7517847). The haplotypes of the tested SNPs were not different between patients and controls. Additionally, analysis according to gender did not show any influence of sex on the association of *IL23R* with Fuchs’ syndrome.

**Conclusions:**

Our results suggested that the rs11209032 AA genotype of the *IL23R* gene may predispose for Fuchs’ syndrome in Chinese patients.

## Introduction

Fuchs’ syndrome is a chronic inflammatory eye disease, which usually presents as unilateral anterior uveitis in young adults [1]. In Chinese patients it is characterized by a mild uveitis with characteristic keratic precipitates, varying degrees of iris depigmentation and occasional heterochromia [2]. Although the etiology of Fuchs’ syndrome is not fully understood, several studies have revealed that genetic factors may be involved in the pathogenesis of the disease [3]. Makley et al. [4] described that monozygotic twins both developed Fuchs’ syndrome. Earlier studies have shown that the frequency of human leukocyte antigen-CW3 (*HLA-CW3*) and human leukocyte antigen-DRW53 (*HLA-DRW53*) was decreased in patients with Fuchs’ syndrome as compared with healthy controls [3]. Additionally, polymorphisms of the cytotoxic T cell antigen (*CTLA*) *4* gene have been found to be associated with Fuchs’ syndrome [5].

The interleukin-23 receptor (*IL23R*) gene is located on chromosome 1p31 and the encoded protein forms a receptor for IL23. *IL23*R is highly expressed in dendritic cells (DC) and has recently been recently identified to be involved in several chronic inflammatory diseases [6-10]. A genome-wide association study has revealed that polymorphisms of the *IL23R* gene are associated with inflammatory bowel disease (IBD) [8]. Several SNPs in *IL23R* have been found to be associated with chronic inflammatory diseases including IBD, psoriasis, and multiple sclerosis [11-13]. Recent studies from our group have shown that *IL23R* gene polymorphisms are associated with Behcet’s disease, an important uveitis entity in China [10]. However, it is not clear whether *IL23R* gene polymorphisms are associated with Fuchs’ syndrome, a disorder possibly mediated by an immune response to a viral (Rubella) infection. In this study we examined the association of *IL23R* polymorphisms with Fuchs’ syndrome and showed an increased frequency of the AA genotype of rs11209032 in these patients.

## Methods

### Patients and healthy controls

A total of 138 patients with Fuchs syndrome and 407 age-, sex-, ethnic-matched healthy controls were recruited from the First Affiliated Hospital of Chongqing Medical University (Chongqing, P.R. China) or the Uveitis Study Center of the Sun Yat-sen University (Guangzhou, P.R. China). The diagnosis of Fuchs’ syndrome was principally a clinical one and generally based on the classic description by Kimura et al. [14] in 1955 adjusted for the typical presentation in Chinese patients [2]. Age and gender distribution are shown in Table 1. The study was approved by the local institutional ethnics committee of The First Affiliated Hospital of Chongqing Medical University. All procedures followed the tenets of the Declaration of Helsinki. The written informed consent was obtained from all the subjects.

**Table 1:** The age and gender distribution of patients with Fuchs’ syndrome and controls.

** **	**Patients with Fuchs’ syndrome**		**Healthy controls**	
Clinical features	Total (n=120)	%	Total (n=407)	%
Age at onset (years±S.D)	37.4±12.3		38.4±12.0	
Range	16–57		24–63	
Sex				
Male	61	50.8	221	54.2
Female	59	49.2	186	45.8

### Genomic DNA extraction and genotyping

Blood samples were collected in EDTA tubes and kept at −70 °C until use. Genomic DNA was extracted by the QIAamp DNA Blood Mini Kit (Qiagen, Hilden, Germany). Amplification of the target DNA in the *IL23R* gene was analyzed by the polymerase chain reaction (PCR) using primers presented in Table 2. Each PCR reaction was performed in 10 μl containing 5 μl Premix Taq (Ex Taq Version; TaKaRa Biotechnology Co. Ltd., Dalian, China), 20 pmoles primers and 0.2 μg of genomic DNA. The PCR conditions were as follows: initial denaturation at 95 °C for 5 min followed by 38 cycles of denaturation at 94 °C for 30 s, annealing at different temperatures (61 °C for rs11209032, 55 °C for rs17375018, and 58 °C for rs7517847) for 30 s, extension at 72 °C for 30 s, and a final extension at 72 °C for 5 min. The SNPs were genotyped by PCR-restriction fragment length polymorphism (RFLP) analysis. PCR products of rs11209032, rs17375018, and rs7517847 polymorphisms were respectively digested with 4 U of XspI (TaKaRa, Dalian, China), BsurI (New England Biolabs, Inc., Ontario, Canada), and Ec0147I (New England Biolabs, Inc, Ontario, Canada) restriction enzymes (Table 2) in a 10 μl reaction volume overnight. Digestion products were visualized on a 3.5% agarose gel and stained with GoldView™ (SBS Genetech, Beijing, China). Direct sequencing was also preformed by the Invitrogen Biotechnology Company (Shanghai, China) using randomly selected subjects (20% of all samples) to validate the method used in this study.

**Table 2:** Primers of *IL23R* SNPs and restriction enzymes used for RFLP analysis.

**SNP**	**Primers**	**Restriction enzyme**
rs7517847	5′-CCTTTCACCTATTCCCAAGGCC-3′	ECO147I
	5′-GGGCCTAGGAGACAGCCCATAA-3′	
rs11209032	5′-CTCCCTACATCACCCTCTTTGCACT-3′	XSPI
	5′-TGATAAGGCAATCCGGTGGTTC-3′	
rs17375018	5′-TTTTTCCCATCTTCTTTCTTAA-3′	BSURI
	5′-CGCCCAGCCCTCTTCTCTAATT-3′	

### Statistical analysis

The Hardy–Weinberg equilibrium (HWE) was tested using the χ^2^ test. We compared the patterns of linkage disequilibrium (LD) of SNPs with the international HapMap subjects using Haploview 4.0. Genotype frequencies were estimated by direct counting. Allele and genotype frequencies were compared between patients and controls by the χ^2^ test using SPSS (version 10.0; SPSS Inc., Chicago, IL). The p values were corrected (pc) using the Bonferroni correction by multiplying the p value with the number of analyses performed. A pc<0.05 was considered significant.

## Results

The Fuchs’ syndrome cohort consisted of 138 consecutive subjects which all belonged to the Chinese Han population (71 males and 67 females). The average age of the patients with Fuchs’ syndrome in the study was 37.4±12.4 years (range: 16 to 57 years). The healthy controls included 407 subjects (221 males and 186 females), with an average age of 38.4±12.0 years (range: 24 to 63 years). The age and gender distribution of the patients with Fuchs’ syndrome and controls are shown in Table 1.

The three SNPs of *IL23R* (rs11209032, rs17375018, and rs7517847) were successfully genotyped in 138 patients with Fuchs’ syndrome and 407 healthy controls. The results showed that the distribution of the genotypes and alleles did not deviate from the Hardy–Weinberg equilibrium (HWE). The distribution of both genotypic and allelic frequencies of the three tested *IL23R* polymorphisms is shown in Table 3. The results showed that the frequency of the rs11209032 AA genotype was significantly increased in patients with Fuchs’ syndrome as compared to controls (p_c_=0.036, OR 1.86, 95%CI 1.21 to 2.86). An increased frequency of the A allele of rs11209032 was also observed in patients with Fuchs’ syndrome as compared with normal controls (56.2% versus 46.7%, p=0.009, OR 1.44, 95%CI 1.10–1.90). However, there was no difference when the Bonferroni correction was performed (p_c_=0.054, n=6). There was no difference concerning the genotype and allele of both rs7517847 and rs1737508 SNPs between the patients with Fuchs’ syndrome and the controls. Haplotype analysis using Haploview software showed no difference between patients with Fuchs’ syndrome and controls. Stratification analysis according to gender did not show an influence of sex on the association of *IL23R* with this syndrome.

**Table 3:** Frequencies of alleles and genotypes of *IL23R* polymorphisms in patients with Fuchs’ syndrome and controls.

**SNP**	**Genotype allele**	**FHC (n=138)**	**Controls (n=407)**	**χ^2^**	**p value**	**pc**	**OR (95% CI)**
rs17375018	AA	9(6.5%)	38(9.3%)	1..36	0.309	NS	0.68 (0.32–1.44)
	AG	62(44.9%)	192(47.2%)	0.209	0.647	NS	0.91(0.62–1.35)
	GG	67(48.6%)	177(43.5%)	1.068	0.301	NS	1.23(0.83–1.81)
	A	80(28.9%)	268(32.9%)	1.471	0.225	NS	0.83(0.62–1.12)
	G	196(71.1%)	546(67.1%)	1.471	0.225	NS	1.20(0.89–1.62)
rs7517847	TT	52(37.7%)	134(32.9%)	1.038	0.308	NS	1.23(0.82–1.84)
	GT	56(40.6%)	198(48.7%)	2.696	0.101	NS	0.72(0.49–1.06)
	GG	30(21.7%)	75(18.4%)	0.727	0.394	NS	1.23(0.76–1.98)
	G	116(42.0%)	348(42.8%)	0.044	0.834	NS	0.97(0.74–1.28)
	T	160(58.0%)	466(57.2%)	0.044	0.834	NS	1.03 (0.78–1.36)
rs11209032	GG	29(20.2%)	111(27.3%)	2.114	0.146	NS	0.71(0.45–1.13)
	AG	64(47.2%)	212(52.1%)	1.345	0.246	NS	0.80(0.54–1.17)
	AA	45(32.6%)	84(20.6%)	8.173	0.004	0.036	1.86(1.21–2.86)
	A	154(56.2%)	380(46.7%)	6.851	0.009	NS	1.44(1.10–1.90)
	G	122(43.8%)	434(53.3%)	6.851	0.009	NS	0.69(0.53–0.91)

In the Table, FHC=Fuchs heterochromic cyclitis; OR=odds ratio; NS=not significant. 95%CI=95% confidence interval; p_c_=corrected p.

## Discussion

The present study was performed to investigate the association of *IL23R* polymorphisms with Fuchs’ syndrome in a Chinese Han population. The results showed that the AA genotype of the rs11209032 SNP was associated with an increased susceptibility to Fuchs’ syndrome. However, there was no difference with regard to the genotypes and alleles of rs7517847 and rs17375018 between patients with Fuchs’ syndrome and normal controls.

Fuchs’ syndrome is a relatively rare uveitis entity [2]. Although the etiology and pathogenesis of Fuchs’ syndrome are not fully understood, several hypotheses including sympathetic lesions, association with ocular toxoplasmosis, vascular abnormalities, viral infections and autoimmunity have been proposed [15,16]. Among these presumptions, a viral infection has been accepted, as supported by the demonstration of local intraocular antiviral antibody production [16-18],Elevatedγ-interferon and interleukin 10, an increased number of CD8^+^ T cells in the aqueous humor and a positive cellular response to retinal S antigen have been reported in patients with Fuchs’ syndrome [19-22]. Several inflammatory diseases have been reported to have genetic background. However, the genetic susceptibility of Fuchs’ syndrome has scarcely been investigated principally due to a lack of sufficient samples from these patients. One hundred and thirty-eight samples, consecutively collected from Chinese Han patients with Fuchs’ syndrome during the past five years, made it possible to analyze the association of polymorphisms of a candidate gene with this disease.

Several strategies can be followed to select a candidate gene in the study of genetic susceptibility to certain diseases. These include the identification of a candidate gene based on Genome-wide association study results or by chosing a susceptibility gene already identified in other related diseases. Alternatively one can chose to study a gene according to its relevant functions that might be functional in the pathogenesis of the disease of interest. In this study, we selected the IL23 receptor (*IL23R*) as a candidate gene principally based on the fact that the interaction of *IL23R* with its ligand IL23 results in an increase of signal transducers and activators of transcription signaling which can consequently promote the production of IL17, a potent pro-inflammatory cytokine already identified to be involved in various chronic inflammatory diseases [23]. It has been suggested that an upregulated production of *IL23R* which is associated with certain SNP alleles in its gene could confer risk for the disease [24]. The association of *IL23R* polymorphisms with several chronic inflammatory diseases or autoinflammatory diseases [11-13,25] also stimulated us to investigate its association with Fuchs’ syndrome in the Chinese Han population. As association studies may be influenced by many factors, the following measures were used to validate the results. The healthy controls and patients within the Chinese Han population were strictly age-, sex-, and ethnically matched. Sequencing of the PCR products was performed in 10% of the tested samples to validate the PCR-RFLP data.

Since there are numerous SNPs in a candidate gene and because only a few SNPs may be relevant in the pathogenesis of disease, it is extremely important to select the relevant SNPs. In this study, we chose rs7517847, rs11209032, and rs17375018 as the tested SNPs are mainly based on earlier studies [10,11,26-28]. The SNPs rs11209032 and rs7517847 were previously found to be associated with diseases in different ethnic populations [11,26,28]. Therefore, we selected them as candidate SNPs. Our results showed that the AA genotype of rs11209032 was positively associated with Fuchs’ syndrome in the Chinese Han population. This result is consistent with earlier findings reported in Crohn’s disease in a German population and in Behcet’s disease in a Chinese Han population [10,25]. We were not able to detect an association between rs7517847 and Fuchs’ syndrome, a result which is in agreement with earlier findings reported in Crohn’s disease in Japan [28]. The third SNP tested in this study was rs17375018, which we recently identified as a susceptibility factor for Behcet’s disease [10]. We did not find an association of this latter SNP with Fuchs’ syndrome, which is consistent with earlier findings observed in VKH syndrome, anautoimmune uveitis entity commonly seen in China [29]. Taken together, these results suggest that Fuchs’ syndrome may have a different genetic background as compared to other uveitis entities.

Like other candidate gene studies, there are several limitations in our study. As the power to detect disease susceptibility genes is influenced by the number of the patient’s samples, the size of patient samples in our study seemed to be relatively small and the patients were only recruited from the Chinese Han population. The results observed in this study need to be confirmed using large sample sizes and in other ethnic populations. Furthermore, it is not clear how the associated SNP, rs11209032, as identified in the present study, exerts its influence on the pathogenesis of Fuchs’ syndrome. More studies are needed to address this issue.

In summary, our study showed that the rs11209032 AA genotype of the *IL23R* gene is positively associated with Fuchs’ syndrome in the Chinese Han population. We did not find an association between the other two tested SNPs of *IL23R*, rs7517847 and rs17375018 and Fuchs’ syndrome in this population.
